# ENIGMA + COINSTAC: Improving Findability, Accessibility, Interoperability, and Re-usability

**DOI:** 10.1007/s12021-021-09559-y

**Published:** 2021-11-30

**Authors:** Jessica A. Turner, Vince D. Calhoun, Paul M. Thompson, Neda Jahanshad, Christopher R. K. Ching, Sophia I. Thomopoulos, Eric Verner, Gregory P. Strauss, Anthony O. Ahmed, Matthew D. Turner, Sunitha Basodi, Judith M. Ford, Daniel H. Mathalon, Adrian Preda, Aysenil Belger, Bryon A. Mueller, Kelvin O. Lim, Theo G. M. van Erp

**Affiliations:** 1 Psychology Department, Georgia State University, Atlanta, GA USA; 2 Tri-Institutional Center for Translational Research in Neuroimaging and Data Science (TReNDS), Georgia State University, Georgia Institute of Technology, Emory University, Atlanta, GA 30303 USA; 3 Imaging Genetics Center, Mark & Mary Stevens Neuroimaging and Informatics Institute, Keck School of Medicine, University of Southern California, Marina del Rey, CA USA; 4 Departments of Psychology and Neuroscience, University of Georgia, Athens, GA USA; 5 Weill Cornell Medicine, Department of Psychiatry, White Plains, NY 10605 USA; 6 Veterans Affairs San Francisco Healthcare System, San Francisco, CA 94121 USA; 7 Department of Psychiatry and Behavioral Sciences, University of California, San Francisco, CA 94121 USA; 8 Department of Psychiatry and Human Behavior, University of California Irvine, University of California Irvine Medical Center, 101 The City Drive S, Orange, CA 92868 USA; 9 Department of Psychiatry and Frank Porter Graham Child Development Institute, University of North Carolina at Chapel Hill, 105 Smith Level Road, Chapel Hill, NC 27599-8180 USA; 10 Department of Psychiatry and Behavioral Sciences, University of Minnesota, Minneapolis, MN 55414 USA; 11 Clinical Translational Neuroscience Laboratory, Department of Psychiatry and Human Behavior, University of California Irvine, 5251 California Ave, Irvine, CA 92617 USA; 12 Center for the Neurobiology of Learning and Memory, University of California Irvine, 309 Qureshey Research Lab, Irvine, CA 92697 USA

**Keywords:** Decentralized, Data privacy, Meta-analysis, COINSTAC, ENIGMA

## Abstract

The FAIR principles, as applied to clinical and neuroimaging data, reflect the goal of making research products Findable, Accessible, Interoperable, and Reusable. The use of the Collaborative Informatics and Neuroimaging Suite Toolkit for Anonymized Computation (COINSTAC) platform in the Enhancing Neuroimaging Genetics through Meta-Analysis (ENIGMA) consortium combines the technological approach of decentralized analyses with the sociological approach of sharing data. In addition, ENIGMA + COINSTAC provides a platform to facilitate the use of machine-actionable data objects. We first present how ENIGMA and COINSTAC support the FAIR principles, and then showcase their integration with a decentralized meta-analysis of sex differences in negative symptom severity in schizophrenia, and finally present ongoing activities and plans to advance FAIR principles in ENIGMA + COINSTAC. ENIGMA and COINSTAC currently represent efforts toward improved Access, Interoperability, and Reusability. We highlight additional improvements needed in these areas, as well as future connections to other resources for expanded Findability.

## Introduction

International neuroimaging collaborations are becoming a staple of clinical neuroscience research, with sample sizes ranging from hundreds to tens of thousands. These studies represent immense investments in research and provide valuable resources for replication of findings and analyses to address novel questions. Within a decade of the first functional magnetic resonance imaging (fMRI) papers, the value of data sharing across institutions has been recognized; e.g., (Governing Council of the Organization for Human Brain Mapping, 2001). Efforts to share data have included many centralized repositories such as the fMRI Data Center (Van Horn et al., 2001, 2005), investigator or institutionally supported databases such as XNAT (Herrick et al., 2016), LONI IDA (Dinov et al., 2010), and COINS (Scott et al., 2011), or more recently the current National Institute of Mental Health’s National Data Archive (https://nda.nih.gov/about/about-us.html). Open data sharing repositories are also growing, including the OpenNeuro, NITRC, and DataLad resources (Halchenko et al., 2016; Kennedy et al., 2016; Poldrack & Gorgolewski, 2017). Decentralized approaches range from the Biomedical Informatics Research Networks (BIRN) federated databases (Keator et al., 2016b; Ozyurt et al., 2010), to attempts to make multiple databases interoperable and queryable across a single interface, such as SchizConnect.org (Wang et al., 2016) and the Neuroscience Information Framework (Gardner et al., 2008). All of these efforts involve efforts to apply FAIR principles, implicitly if not explicitly—getting neuroimaging data and their associated behavioral and other data out of the “desk drawer”, and making them Findable, Accessible, Interoperable, and Reusable, to different degrees (Wilkinson et al., 2016).

FAIR principles are being explicitly adopted and supported at many levels nationally and locally, even in neuroimaging research. For example, OpenNeuro and DataLad provide standardized metadata regarding data provenance, as well as unique identifiers for datasets. It is not yet common practice to fully implement FAIR recommendations, however, through providing persistent and unique identifiers for datasets or standardizing the metadata formats or access, for example. In this paper we discuss how the international ENIGMA consortium practices together with the software platform COINSTAC are addressing FAIR principles for the neuroimaging community. We include an example of a decentralized COINSTAC analysis that examines sex differences in symptom severity in schizophrenia. We assess the current FAIR capabilities of their practices, and make recommendations for improved FAIR compliance.

## ENIGMA: Promoting Findability, Accessibility, Interoperability, and Reusability

The Enhancing Neuroimaging Genetics through Meta-Analysis (ENIGMA) consortium is a data integration initiative coordinating large-scale analyses of brain imaging, genetics, clinical and behavioral data, across 45 countries (Thompson et al., 2020). The consortium, founded in 2009, has grown to include over 2,000 scientists working together on questions in neurology, psychiatry, and brain development. Crucially, ENIGMA lowered the sociological and technical barriers to entry, by initially asking neuroimaging researchers worldwide to collaborate on “prospective meta-analyses” – in other words, coordinated analyses of existing data, where there was no requirement to centralize individual-level data at any one site (Bearden & Thompson, 2017; Ching et al., 2020a, b; Thompson et al., 2014, 2020). The neuroimaging community has responded positively, with analyses of massive datasets, from studies that individually may lack the statistical power to definitively answer certain questions, but when combined have resulted in some of the largest studies in neuroimaging to date; e.g., (Hibar et al., 2018; Kong et al., 2018; Renteria et al., 2017; van Erp et al., 2016, 2018). In the first meta-analyses performed by ENIGMA, participating sites analyzed their data using the same agreed-upon, harmonized methods and statistical models, and returned summary results to a central site for a meta-analysis.

Through concerted efforts to bring in researchers from around the world, ENIGMA has grown into a massive collaborative effort organized around over 50 working groups focused on clinical studies, methodological approach development for a range of imaging modalities (MRI, diffusion imaging, resting-state fMRI and EEG), and genetic as well as epigenetic analyses. The methods have expanded to include data sharing and centralized aggregation for some analyses, allowing for a “mega-analysis” rather than meta-analyses, e.g., (Boedhoe et al., 2018; Ching et al., 2020a; Hoogman et al., 2020; Zugman et al., 2020), though the data aggregation approach limits participation by sites who are not allowed to share individual data points due to local regulations and ethical concerns. In short, the consortium has supported coordinated immensely powerful analyses that are entirely distributed, or entirely centralized, as well as combinations of each approach.

### Findability

ENIGMA has been a wildly successful data analysis consortium, even though the datasets that are accessed for ENIGMA analyses are at the present largely not machine findable as proposed by the FAIR principles. The FAIR findability principle requires that data include a globally unique and persistent identifier and rich associated metadata, which are both registered or indexed in a searchable resource. More generally, a spectrum of “findability” for datasets to allow for a range of FAIR solutions ranges from datasets and their metadata being directly linked to identifiers, to a link for either the dataset or metadata without the elements of the datasets being available (Mons et al., 2017). In each case, the identifier is required, as a foundation for FAIR compliance (Juty et al., 2020). Particularly for groups outside of the United States, data are most often not in publicly available repositories, and some types of data sharing may be limited by national and international regulations such as the General Data Protections Regulations in the European Union (GDPR; (Union, 2016)). Associating an identifier with a dataset or metadata generally doesn’t happen at the level of an individual investigator’s datasets inside their home institutions’ firewalls, which ENIGMA is working with. But with the addition of a unique and persistent identifier to index the existence of these datasets, these datasets could fall under Mons et al.’s label of “FAIR-findable”.

Lacking the adoption of these identifiers, ENIGMA’s approaches to finding data are utilitarian. The datasets that are being included in ENIGMA analyses are Findable in some cases through organized semantic wikis such as NITRC (nitrc.org), NIF (neuinfo.org), or ODS (www.organicdatascience.org), but largely through calls for participation in any given projects. Finding the data for completely new ENIGMA projects at this time often occurs via literature searches, referrals by ENIGMA consortium members, and advertising to the wider community; e.g., via the ENIGMA website and conference presentations. Finding the data used in specific ENIGMA publications or projects is helped by detailed supplemental material in publications, with the cohort descriptions as well as the means to access them via working group chairs and cohort investigators. ENIGMA has used many formats to help researchers learn about ongoing projects overall and to provide access to the data and collaborative network. Beginning with an initial email calling for a collaborative genome-wide scan analysis of structural imaging data as the first project and extensive personal communication including multiple conference presentations and invited seminars, ENIGMA investigators now hold regular workshops around the world to engage researchers internationally in learning the methods used or in joining or leading new projects’ analyses.

### Access

to the data comes along with Finding it. ENIGMA is not a data sharing resource, and the notion of access to “the ENIGMA data” is misconstrued. Joining ENIGMA involves joining one or more working groups, usually through signing their Memoranda of Understanding (MOUs) and joining in on teleconferences and projects. Access to the data for an analysis is managed through the investigators who have access to the data personally. For example, the ENIGMA Schizophrenia working group has published two studies that both used the same meta-analysis technique: we leveraged a structural image processing using the FreeSurfer software (Dale et al., 1999; Fischl, 2012; Fischl et al., 2002) which is the earliest processing pipeline that ENIGMA groups agreed upon. FreeSurfer output was then analyzed using standard R scripts (R Development Core Team, 2020) to assess the effects of diagnostic status (case/control) across the quality-controlled FreeSurfer-derived brain measures. Each site ran the ENIGMA FreeSurfer protocols on their data, and then ran the R scripts on the output to perform the same analysis. The analysis results at each site were then sent via email or uploaded to the organizing site for meta-analysis. Using this highly distributed approach, the analysis of case/control differences in subcortical volumes included data from 5,000 individuals, and the subsequent analysis of cortical measures included almost 10,000 individuals (van Erp et al., 2016, 2018). Sites that were able to run the FreeSurfer processing and the R scripts, and return the results, were included in the analysis, as were sites that could share either the raw images or the individual FreeSurfer brain measures for inclusion in the analysis by the organizing site. In another analysis, individual subject level data FreeSurfer were shared to compare meta- and mega-analysis approaches (Radua et al., 2020). Other agreed-upon protocols within ENIGMA for diffusion tensor imaging analysis, brain region shape analyses, and genetic analyses have been tested, published on, and made available to the research community (http://enigma.ini.usc.edu/protocols/).

### Interoperability

in these analyses, as in the diffusion tensor imaging analyses and other distributed analyses, comes from ENIGMA investigators agreeing to organize their data in the same way for a project’s analysis. Each different project which performs a meta-analysis, whether it is analyzing cognitive correlates of white matter measures in schizophrenia or genetic effects on subcortical volumes generally, develops their own analysis plans and requirements as tuned for their particular questions. For example, in the genetic analyses of subcortical volumes, participating sites discussed and agreed on allowable imaging segmentation software to use, quality assurance techniques to use, and how to organize the subcortical volumes into a consistent spreadsheet format so that each site’s data were consistently arranged and the same analysis performed. They also agreed on imputation techniques, quality control steps, software to use and analysis scripts for the genetic data analyses. These agreements on data processing and organization are not “standards” that are intended for universal use, only for a given project—with subsequent projects doing new analyses of other imaging types or genetic data, the choices that worked well in previous projects can be kept, while improvements or completely new directions can be included. It is important to note that it may become more efficient to adopt the developments of more generalizable neuroinformatics data sharing standards (e.g., www.repronim.org) in future projects. This will require some extra effort, and thus is most likely to succeed when driven by a question that requires data not yet generated by the adopted system for data organization.

For structural analyses, the FreeSurfer output format and directory structure have been the same for decades, and ENIGMA’s cortical and subcortical analyses have leveraged that directory structure, in pulling together the brain measurements into csv (comma separated value) files that are the same across sites. The non-imaging data such as age, sex, diagnostic status, and other measures, are handled independently by each site and arranged into an agreed-upon format for the analyses to complete. The analyses are also agreed upon ahead of time, including various R scripts regression models, which perform the analyses using the same commands and save the same output across sites. This includes all the brain regions being analyzed in all the models with and without various confounding variables. The naming conventions for the output often are explicitly used to read each site’s results and perform the meta-analyses; often, hundreds of meta-analyses. Quality control of the results is critical, to ensure individual sites’ data are not miscoded or mis-analyzed in some way that would invalidate the meta-analysis. This is done at each site, but also often by the central site, which can compare across sites’ results and range of measures to identify sites whose output is unusual.

With these agreements for access and interoperability of the data, however, ENIGMA’s approach has been wildly successful in making data Reusable. Many of the clinical working groups have published meta- or mega-analyses of subcortical and cortical data in their clinical disorder of interest (Boedhoe et al., 2020; de Zwarte et al., 2019; Hibar et al., 2018), diffusion tensor imaging analyses have also flourished (Holleran et al., 2020; Kelly et al., 2018), and more nuanced analyses of symptom severity and environmental effects have also been successful (Walton et al., 2017, 2018). At present, reusability of data is again mediated by humans, by investigators agreeing to participate in various projects, or to share their data in common repositories. Because ENIGMA working groups adopt the same image analysis and quality control procedures across working groups, generated data can also be re-used for analyses to address questions that span multiple working groups such as imaging genetics (Enhancing Neuro Imaging Genetics Analysis et al., 2012; Hibar et al., 2015), cross-disorder comparisons (Boedhoe et al., 2020), brain laterality results (Kong et al., 2018), and analyses of changes across the lifespan (Frangou et al., 2019).

## COINSTAC: Promoting Findability, Accessibility, Interoperability, and Reusability

The Collaborative Informatics and Neuroimaging Suite Toolkit for Anonymous Computation (COINSTAC) is a software platform that allows for decentralized analyses (Plis et al., 2016). The goal is for the data to remain at their source location, behind their institutional firewall, and yet to be available for inclusion in cross-institutional integration and analyses. The development of COINSTAC is particularly motivated by the need to protect participant privacy and confidentiality, for datasets that cannot be shared due to identifiability concerns (e.g. rare genetic disorders) or legal or regulatory restrictions (Sarwate et al., 2014). To this end, COINSTAC includes a range of privacy-preserving features. All of the algorithms in COINSTAC are designed from the ground up to only share derived data, not original data, from a site. The fact that individual-level data is never shared with a remote site provides a basic level of protection. Furthermore, for particularly sensitive data, a pipeline developer can leverage algorithmic privacy such as differential privacy algorithms (Sarwate et al., 2014).

The COINSTAC platform allows for federated or decentralized data analysis via sharing of analysis pipelines and peer to peer communication of partial results, updated models, etc., as needed for federated learning, for example. The COINSTAC client software works via download of analysis pipelines generated by a group that leads a consortium. Investigators can design and specify the analysis they want to run (e.g., group differences in brain region volumes after controlling for age and intracranial volume, or a machine-learning classification analysis on gray matter images), which then also determines the needed data for each subject included in the analysis (e.g., gender, age, intracranial volume, and the desired brain region volumes; or the gray matter images along with needed subject and scanner information). Once the analysis is designed, the investigator can start a consortium. Other investigators who have COINSTAC installed on their systems can then join the consortium with a click of a button, which initiates a download of the needed analysis pipeline in a Docker container (Merkel, 2014). The data for the consortium analysis then needs to be “mapped”, through identifying the data files on their local file system with the covariates of interest (e.g., gender, age, intracranial volume, and brain region volumes) and paths to local gray matter images and other relevant data, and associating columns in those files to the needed variables for the computation pipeline. Once the data are mapped, the individual pipeline can run automatically at each participating site, with the needed summary or intermediate results passed back and forth as needed to perform the initial analysis and meta-analysis, or for the machine learning analysis to complete. This process improves interoperability between datasets. COINSTAC is hosted at GitHub (https://github.com/trendscenter/coinstac), along with example instructions for its use. The software is shared using an MIT license, which can be found at (https://github.com/trendscenter/coinstac/blob/master/LICENSE).

### Findability and Access

The COINSTAC system is currently in between a fully automated system that can identify any arbitrary needed variables from all the data a site has without human involvement, and a fully manual system like ENIGMA that requires the site personnel to reorganize and extract their needed data for each analysis. COINSTAC is open to anyone who installs the software. However, like ENIGMA, it is not a data repository. COINSTAC is still implementing methods for finding data, or adding identifiers to the pipelines, datasets, or results, and developments toward those ends are described in more detail below. In COINSTAC, similar to within ENIGMA, a user can start a consortium, create a pipeline for an analysis, map their own data as needed for that analysis, and run the analysis either on their own data only or on the data of other users and sites that have joined the consortium and mapped their data as well. Methods to advertise a new consortium among registered COINSTAC users, as well as methods for giving blanket approval for one’s data to be included in any relevant analysis, are in the development plans.

### Interoperability and Reusability

Consistent data organization is a key point, that in some ways COINSTAC’s interface was designed to address. The COINSTAC interface allows mapping of particular variables, so that idiosyncrasies and inconsistencies across research labs can be avoided. For example, in a standard regression of age and gender against hippocampal volume, in which data are in a spreadsheet, one site might label age as “Age” while another group labels it as “AgeinYears” or even “V1” or any arbitrary string. COINSTAC includes a “data mapping” procedure in which the site team joining a consortium can indicate where their data are, and which variables are which for the needed analysis. This minimizes the data handling requirements for the site joining a consortium, without requiring a standard naming scheme or data organization, for example, or requiring that people re-type in their data to a webform either once or for every study. It allows generalizability from one analysis pipeline to another, so that there is not one fixed data naming scheme or set of variables that all studies are expected to conform to, and evolving quality assurance steps can be added to new pipelines as needed, rather than depending on a priori input validations.

### Example COINSTAC Analysis

To demonstrate the use of COINSTAC in a multi-site consortium, we ran a meta-analysis examining sex differences in negative symptom severity in individuals with schizophrenia using data collected by the FBIRN (Function Biomedical Informatics Research Network). The FBIRN data were collected at seven institutions with cross-site clinical harmonization, including multiple symptom scale assessments. Each site’s dataset was run as a separate COINSTAC site and combined in a COINSTAC consortium. We present the results of the sex differences in negative symptom factors here as a simplified example; more complex analyses relating symptom severity to imaging data are being performed but are beyond the scope of this paper.

**Fig. 1:**
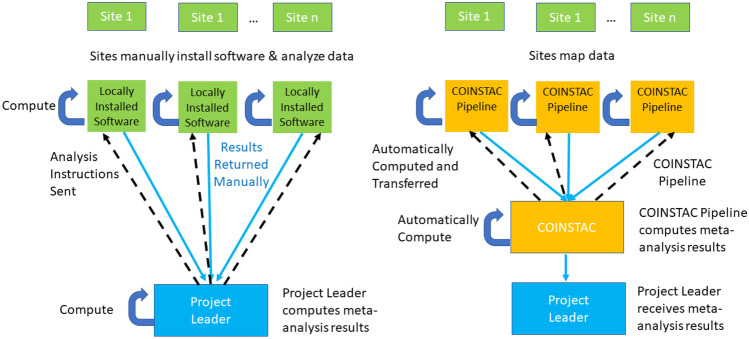
The example analysis workflow as originally implemented for ENIGMA meta-analyses (left) and as implemented with COINSTAC (right). For more detail see text

In Fig. 1 we show the workflows for how ENIGMA would implement this meta-analysis without COINSTAC (left) and how it is implemented within COINSTAC (right). The participating sites are shown along the top, and each have the relevant data for the analyses. Without COINSTAC, the project leader (at the bottom), develops the analysis instructions or scripts, sends them to all sites (dotted arrows), who locally install any needed software and implement the analyses, and return the results to the project leader (either via email or upload to a shared location). The project leader aggregates the results and runs the meta-analysis script. With COINSTAC (right), the project leader sets up the pipeline for the local analyses and meta-analysis within COINSTAC. The sites join the consortium within COINSTAC, map the locations of their data as needed for the analysis, and COINSTAC runs the analysis pipeline on their data on their machines, aggregates the results, and performs the meta-analysis, returning the results to the project leader. If this were a more complex analyses, e.g., an iterated algorithm for data decomposition or federated learning, COINSTAC would implement all the parameter passing and model updating, etc.

In this example, we analyzed the Schedule for the Assessment of Negative Symptoms (SANS; (Andreassen, 1984)) from 185 participants (139 males, 46 females) with schizophrenia from seven sites. Confirmatory factor analyses, including hierarchical models, of negative symptom data have identified two broad and five subordinate negative symptom domains (Ahmed et al., 2019; Strauss et al., 2018, 2019a, b). Historically, men with schizophrenia are reported to have more severe negative symptoms than women with schizophrenia (Abel et al., 2010; Ahmed et al., 2014; Gur et al., 1996; Maric et al., 2003), though these effects have not been examined for individual negative symptom domains.

## Methods

We developed R scripts to read the clinical and demographic data, to calculate five factor model and two-factor model scores from SANS item data, and to regress these scores against gender. The five-factor model generates scores for Anhedonia, Asociality, Avolition, Blunted Affect, and Alogia, and the two-factor model generates scores for Motivation/Apathy or MAP, which is a weighted combination of Anhedonia, Asociality, and Avolition, and Expressiveness or EXP, which is a weighted combination of Blunted Affect and Alogia. Each site had the SANS and gender data in a standardized comma separated value (csv) file, though those spreadsheets could be in any directory on the local system, as the user identifies the needed files during data mapping. The analysis calculated the total negative symptom scores, the five and two factor scores based on the SANS for each subject at each site. The relationship between self-reported gender (M or F) and these scores were calculated in R using the lm function, and each site’s results were then combined in a meta-analysis. The mixed-effect meta-analysis was performed using R’s metafor package including site as a random effect and gender as fixed effect.

## Results

**Fig. 2:**
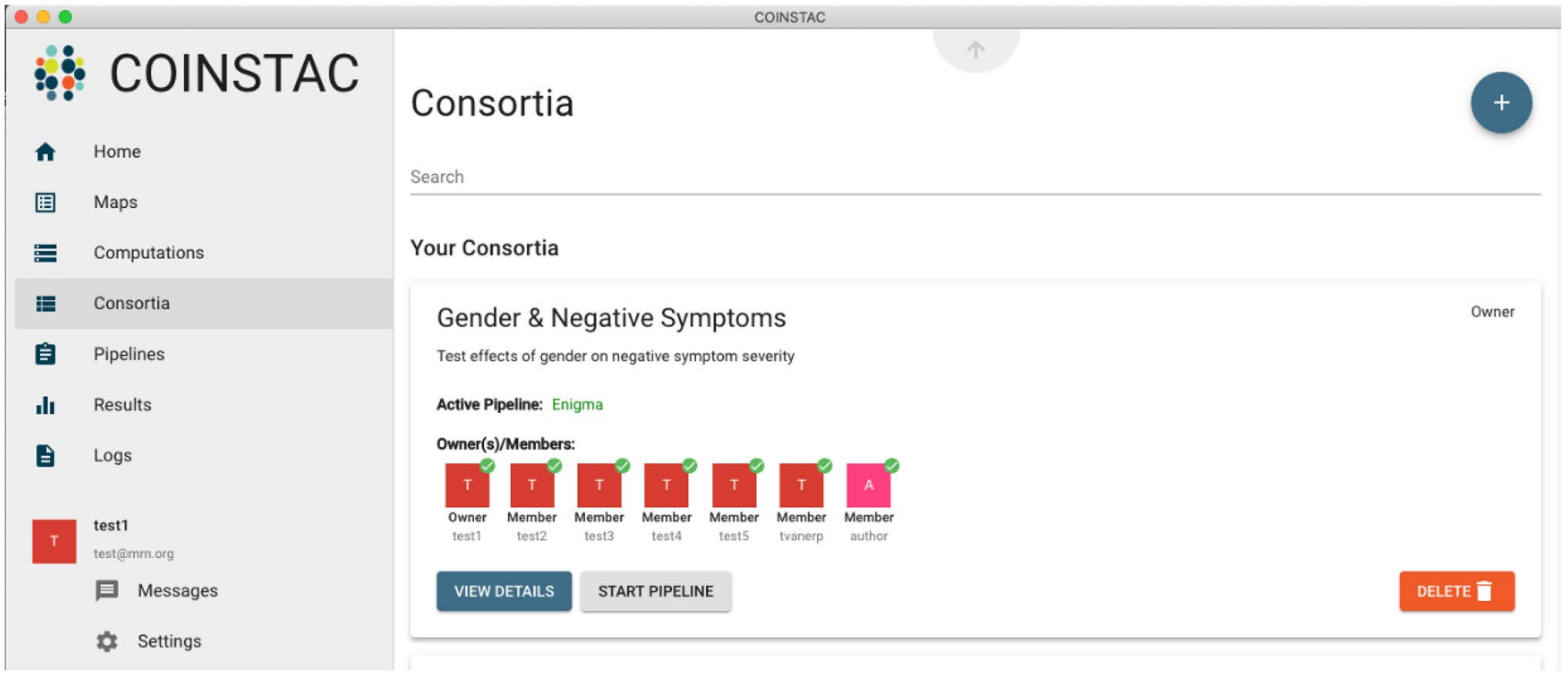
Example COINSTAC Consortium. A screenshot of the consortium set up with the members having joined and mapped their data as needed for the consortium analysis pipeline

**Fig. 3:**
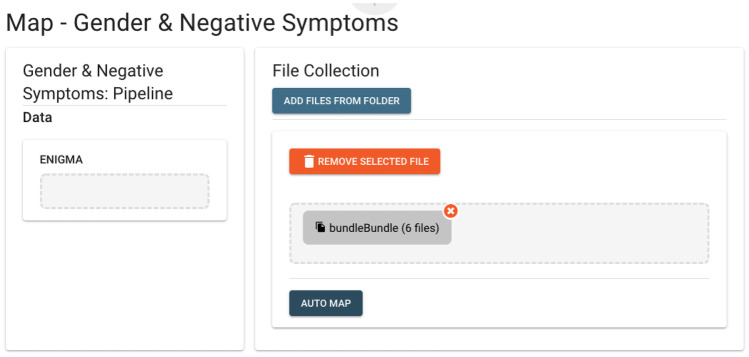
Example Data Mapping. For this consortium analysis, data mapping consisted of identifying the needed files, which were then grouped as a bundle for use in the analysis. Data mapping does not move the files, but sets up the communication needed for the analysis pipeline to find the needed files when it runs locally

In Figs. 2 and 3, the user “test1” started a consortium named “Gender & Negative Symptoms” in order to run the analysis pipeline named “ENIGMA”. A project leader can start a new consortium via the “Consortia” item in the main COINSTAC menu (Fig. 2). The leader then names the consortium, selects an analysis pipeline, and members can then join the consortium. Here, six other groups joined as members in the consortium. After logging into COINSTAC, consortium members download a dockerized version of the consortium-specific analysis pipeline to their local COINSTAC instances. They then map their data files for analysis under “Maps” in the main menu, which in this case included pre-arranged csv files with the relevant clinical and demographic data (Fig. 3). Once every member has mapped their data, the consortium owner launches the pipeline. In this meta-analysis example, the pipeline first ran analyses at each site on the local data, then uploaded the analysis results to COINSTAC central, where the meta-analysis was run. The local results from each site plus the meta-analysis results were then transferred to the project leader.

The COINSTAC platform was used to initiate the consortium, provide a pipeline of the needed R version, libraries, and the R scripts to all sites, to start the analyses at all sites, transfer the results of each analysis when done, and then to automatically perform the meta-analysis, and provide the results to the consortium owner at completion. One major advantage of using the COINSTAC is that apart from downloading and running the COINSTAC application and Docker, no local software installations are needed for COINSTAC-initiated decentralized data analyses.

**Fig. 4:**
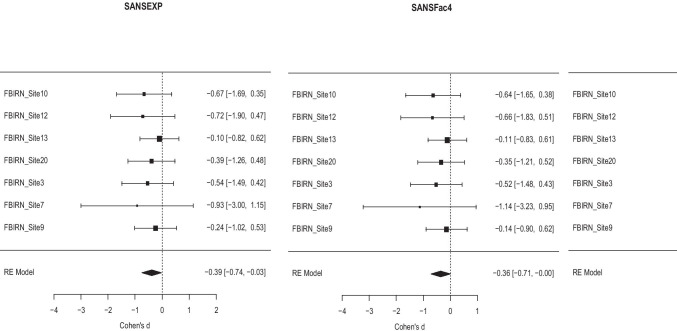
Forest plots for Meta-analysis of Gender Differences in EXP (Expression), and associated Blunted Affect (Fact4), and Alogia (Fact 5) Negative Symptom Domain Factors

The meta-analysis was performed using the results from seven datasets from the FBIRN consortium; see Table 1 for the site information. All subjects were stable on antipsychotic medication, and had a minimum of a one year’s diagnosis; for inclusion and exclusion criteria please see (Turner et al., 2013). The meta-analytic results of gender effects (M > F) on the various SANS factors and total scores are shown in Table 2. Generally, women’s symptom scores were less severe than men’s, though only the EXP factor and its components Blunted Affect and Alogia reached meta-analytic *p* values less than 0.05. The forest plot of these effects across site is shown in Fig. 4.

**Table 1:** Demographics of the participating sites’ samples as shown in Fig. 2, including the site, the number of subjects with SZ, the number of self-reported male and female (M/F), the mean age in years, the mean duration of illness in years (DOI), and the means SANS total score for the sample

**Site**	**N**	**M/F**	**mean age (range)**	**mean DOI (range)**	**mean SANS Total (range)**
test1	28	22/6	35.8 (22–53)	12.3 (1–27)	19.6 (1–61)
test2	15	14/1	41.7 (23–58)	19.3 (3–41)	29.5 (9–54)
test3	35	26/9	44.5 (20–60)	23.3 (2–40)	15.9 (2–63)
test4	31	26/5	37.0 (21–62)	15.3 (3–49)	20.4 (0–48)
test5	15	10/5	37.1 (21–51)	16.3 (2–31)	20.3 (0–44)
Test6 (tvanerp)	31	18/13	36.6 (19–56)	15.1 (2–48)	18.6 (4–45)
Test7 (author)	32	25/7	39.3 (18–60)	18.5 (1–39)	19.4 (0–53)

**Table 2:** For each score (Total, MAP, EXP, and the five factors), the meta-analysis estimate of Cohen’s *d* for the gender effect, the standard error, the z, *p*, and effect size confidence interval lower bound (ci.lb) and upper bound (ci.ub)

	**Cohen’s d**	**SE**	**z**	**p**	**ci.lb**	**ci.ub**
**Total SANS**	-0.29	0.18	-1.62	0.10	-0.64	0.06
**MAP**	-0.05	0.17	-0.30	0.76	-0.40	0.29
**EXP**	-0.39	0.18	-2.14	0.03*	-0.74	-0.03
**Anhedonia**	0.094	0.18	0.52	0.61	-0.26	0.44
**Asociality**	0.049	0.18	0.22	0.83	-0.31	0.39
**Avolition**	-0.26	0.18	-1.45	0.15	-0.61	0.09
**Blunted Affect**	-0.36	0.18	-1.99	0.047*	-0.71	-0.004
**Alogia**	-0.36	0.18	-1.97	0.04*	-0.729	-0.001

* denotes *p* < .05 for the EXP factor score and its two subdomain factors

## Discussion

The main objective of this study was to demonstrate the use of the COINSTAC platform in a multi-center federated data analysis setting such as performed in the ENIGMA (Enhancing Neuro-Imaging Genetics through Meta-Analysis) consortium and to assess how the combination of COINSTAC and ENIGMA can facilitate the use of FAIR data principles (see Table for current status). To this end, this study ran a COINSTAC meta-analysis exploring gender differences in negative symptom severity based on recently published two and five factor negative symptom domain analyses. We found that total negative symptom severity was not significantly higher in males compared to females (*P* > 0.05). However, on closer examination, the EXP (expression) but not the MAP (motivation and pleasure) factor score, and its corresponding 5-factor model negative symptom sub domains of Blunted Affect and Alogia, were more severe in men compared to women with schizophrenia. To our knowledge, is the first report of gender differences in these symptom factor scores in schizophrenia that may at least provide an initial external validation for the 2-factor model of negative symptoms in psychosis.

### Scope of ENIGMA + COINSTAC

It is critical to note that the collaboration of ENIGMA and COINSTAC does not create a data repository along the lines of the NIMH Data Archive, or Open Neuro, that a user can request data from. It is not a data management and sharing system for neuroimaging studies like XNAT or COINS. COINSTAC is not a pipeline design framework for centralized data like the LONI IDA or CPAC, for example. It is not a deep-learning AI platform for neuroimaging such as Clara (https://developer.nvidia.com/clara). It is an open-source platform designed for implementation of a broad range of decentralized neuroimaging analyses for datasets which do not allow direct access for sharing. Since ENIGMA was also designed to work within data sharing restrictions, the two efforts are collaborating to push the application of COINSTAC methods to ENIGMA analyses, both to facilitate the meta-analyses and to allow for federated learning approaches.

The push to move ENIGMA + COINSTAC toward FAIR principles is summarized in Table 3. The table lists whether the current COINSTAC system includes methods for addressing the principle, whether it does not apply, whether it facilitates methods for addressing it (e.g., COINSTAC does not provide Digital Object Identifiers (DOIs) for datasets, but points users to Zenodo and other sites, and will store the DOI if provided), or if the methods for addressing it are in development. Table 3 lists the FAIR principles and how the current ENIGMA + COINSTAC addresses these not only for datasets, but also to analysis pipelines, results, and the overall projects. Metadata standards for describing each of these are still in development, and likely to remain so as more detailed descriptions become possible. We discuss each of these below.

**Table 3:** FAIR principles’ status through ENIGMA + COINSTAC

**FAIR principle**	**Consortium project**	**Datasets**	**Analysis Pipelines**	**Results**
F1. (meta)data are assigned a globally unique and persistent identifier	In development	Facilitated	Addressed	Addressed
F2. data are described with rich metadata (defined by R1 below)	N/A	Facilitated	Facilitated	In development
F3. metadata clearly and explicitly include the identifier of the data it describes	Addressed	Facilitated	Addressed	Addressed
F4. (meta)data are registered or indexed in a searchable resource	In development	Facilitated	Addressed	Addressed
A1. (meta)data are retrievable by their identifier using a standardized communications protocol	In development	In development	In development	In development
A1.1 the protocol is open, free, and universally implementable	Addressed	Addressed	Addressed	Addressed
A1.2 the protocol allows for an authentication and authorization procedure, where necessary	Addressed	Addressed	Addressed	Addressed
A2. metadata are accessible, even when the data are no longer available	Facilitated	Facilitated	In development	In development
I1. (meta)data use a formal, accessible, shared, and broadly applicable language for knowledge representation	In development	In development	In development	In development
I2. (meta)data use vocabularies that follow FAIR principles	In development	In development	In development	In development
I3. (meta)data include qualified references to other (meta)data	In development	In development	In development	In development
R1. meta(data) are richly described with a plurality of accurate and relevant attributes	In development	In development	In development	In development
R1.1. (meta)data are released with a clear and accessible data usage license	Addressed	Facilitated	Addressed	Addressed
R1.2. (meta)data are associated with detailed provenance	N/A	Facilitated	In development	In development
R1.3. (meta)data meet domain-relevant community standards	In development	In development	In development	In development

### Findability improvements

This decentralized analysis example highlights several strengths and weaknesses of the approach. Standards for identifying existing consortia, the datasets used in their analyses, the analysis pipelines, and the results need to be integrated as they develop. Within COINSTAC, project consortia do not yet have DOIs (though they have UUIDs in the COINSTAC database of projects), and datasets used in an analysis can have DOIs such as those from Zenodo included. We are facilitating the acquisition of DOIs by creating a community on Zenodo dedicated to COINSTAC datasets (https://zenodo.org/communities/coinstac/) and have seeded it with a sample dataset (https://zenodo.org/record/5425443). We have also developed a Discovery feature which stores Brain Imaging Data Set (BIDS) format data descriptions when those are available, and BIDS format specifications includes DOIs for the datasets (Gorgolewski et al., 2017). This allows for searching within the datasets that have these descriptors. We are in the process of creating an API that will allow other platforms and websites to search for datasets on COINSTAC, thereby improving interoperability as well. Methods for improving findability could certainly include searches of available repositories such as DataLad’s automated searches for available data, or searching the NDA or SchizConnect data repositories for the needed data, and including the relevant datasets from one of those resources in the consortium as another “site”.

As these kinds of efforts become more standardized, it would be beneficial to have COINSTAC be able to access and query for relevant datasets for new consortia or analysis pipelines. COINSTAC Discovery capabilities currently include the functionality for making existing consortia searchable, e.g., providing such an identifier for the consortium and its analysis pipeline, along with contact information for the consortium organizer. This will aid in making existing consortia and their analyses findable, both for replication and reproducibility, without compromising data access restrictions. The results of an analysis are local to the consortium lead site, but can be uploaded outside of COINSTAC, for example statistical brain maps to NeuroVault (Gorgolewski et al., 2016b). The pipelines included in COINSTAC are built as Docker images and have a unique URL on the COINSTAC Github site, though including rich semantic standard metadata describing the pipelines needs to be an ongoing collaboration with the Neuroimaging Data Model (NIDM), which focuses on the description of experiments, analyses, and results (Keator et al., 2016a). COINSTAC has included an initial feature to automatically generate NIDM results for our VBM regression pipeline; generalizing this to arbitrary pipelines is an ongoing effort. We are in the process of automatically mirroring new GitHub repository releases of COINSTAC pipelines on Zenodo, which will provide them with DOIs. The ENIGMA Organic Data Science platform is also planned as a human-usable interface for building and tracking consortia (McMahon et al., 2018). Within ENIGMA, the Organic Data Science platform includes a centralized index of analyses that includes searchable metadata to facilitate comparing methods and findings across different working groups and domains. These plans will need to link to standard formats for identifiers, rich metadata, and detailed provenance regarding the analyses and the vocabularies used to describe the analyses, as noted below.

### Access

The COINSTAC software is freely available, and access to the interacting COINSTAC network does include user accounts with authentication, encryption, and permission-based access. Once the data are mapped for an analysis pipeline within a consortium, COINSTAC accesses only the data that have been mapped, and only for the consortium analysis pipeline that was agreed to. Access is not automatic simply because an investigator has installed COINSTAC; data has to be mapped for a particular consortium and analysis. In effect, COINSTAC acts similarly to a Data Usage Agreement that says specific data will be shared only for a particular project or analysis plan, and no attempts at de-identification or other analyses will be made other than what is in the pipeline. Thus access is protected through local data analysis without upload of individual subject data to a central repository, or transfer of individual subject data between institutions. Methods for pre-approval are certainly desirable, so that data that are unrestricted could be automatically included from a site with a COINSTAC installation.

### Interoperability

The metadata vocabularies needed for the Interoperability and Reusability principles for consortia, datasets, analyses, and results are in early stages, and their use will need to be developed, as noted in Table 3. The decentralized analysis presented in this paper highlighted the usefulness of standards for data organization and management, as the informal arrangement of data in the csv files constituted the “standard” for data organization for this analysis, making the analysis immediately possible. The structure applied here is clearly not generalizable to other projects or datasets, however, as the data organization is set up only for a specific study and has meaning only within this analysis. The challenge is to use a more generic vocabulary, meaningful beyond the confines of a specific current study, with the goal of making scientific data interoperable and reusable in a larger context (Burns & Turner, 2013; Turner et al., 2015). Other imaging-based pipelines implemented in COINSTAC leverage the BIDS structure, so that different imaging modalities are clearly marked and relevant imaging parameters are stored in consistent ways (Gorgolewski et al., 2016a, 2017). A great deal of work has gone into clarifying the underlying structure of the relevant imaging data that can be identified and compared across datasets, to design BIDS to represent the needed information robustly. Imaging pipelines can take advantage of having data in BIDS format, to implement robust analyses across large and diverse datasets.

The non-imaging data, such as the clinical assessment scores, behavioral and demographic data or other relevant measures, is not covered by BIDS. Organizing the data and ensuring that they are coded consistently from one lab to another are perennial problems in biomedical data sharing, which many efforts have attempted to address, e.g. (Bandrowski et al., 2016; Chen et al., 2018; Keator et al., 2009; Ozyurt et al., 2010, 2016; Turner & Laird, 2012; Zaslavsky et al., 2016). Agreeing to organize the data as needed by the NIMH Data Archive, for example, or for other large-scale efforts with some agreements on terminology, acceptable values, and organization, would allow more generalizable data structures for various COINSTAC consortia and analysis pipelines, while working toward developing a fully realized interoperable annotation of the data in keeping with FAIR principles. ENIGMA does not at the moment use that approach, though as the different ENIGMA working groups are collaborating for cross-disorder analyses, agreements about the clinical and demographic data coding and arrangement are taking place as needed, e.g. (Kochunov et al., 2020; Navarri et al., 2020).

### Reusability

The analysis container used in this example analysis ran a simple regression and meta-analysis using containerized R code. This was a simplified example, and pipeline developers are not limited to the options included here. Including the analysis for a COINSTAC consortium as a container ensures that the analysis is the same at all sites; that it is not dependent on the individual site investigator installing arbitrary software, or implementing the analysis; and that there is a record for reproducing the analysis as needed, as the pipelines can be stored and re-used. While this does not address the reusability of the datasets per se, it does address issues of reproducibility of the analysis, as the same analysis can be implemented again or applied to an independent group of participating sites in an identical manner.

While in this study we implemented a simple regression based on regions of interest, more complex algorithms have been developed to work in the decentralized environment; the full list of algorithms for COINSTAC is available publicly (COINSTAC, 2020) and includes preprocessing, voxel-based approaches and can take various types of input including structural, functional, and diffusion MRI data. Building upon these developments, a recent voxel-based morphometry analysis of over 2000 datasets collected in Europe, India, and China was performed to study brain structure relationships with age, body mass index, and smoking (Gazula et al., 2019). The regression analysis, however, is a single communication step between the consortium organizer and the participating sites: Each site runs an analysis and sends it to the central site for meta-analysis.

With COINSTAC, if the analysis is an iterative analysis as in federated learning, each site would run an analysis, and then the central site would aggregate the results, update perhaps several parameters, and have each site run an updated analysis, and so on until the algorithm converges (Li et al., 2019; Mothukuri et al., 2021). This iterative process has to be automated, so that machine learning and latent variable analyses can be conducted in a decentralized environment. Currently decentralized versions of iterative regression, independent component analysis for static and dynamic network connectivity analyses, support vector machines, and distributed t-stochastic neighbor embedding (t-sne) for visualization are all available through COINSTAC (Gazula et al., 2018; Saha et al., 2017; Plis et al., 2016; Sarwate et al., 2014; Baker et al., in press; Saha et al., 2020). In some cases a shared reference data set is leveraged, or testing/training configurations are incorporated. An initial deep neural network approach has been developed as well, with the addition of GPU support for computational efficiency in process (Lewis et al., 2020). The strength of a COINSTAC implementation is allowing exactly these iterative solutions which would not be available manually without data aggregation in a single institution or cloud environment.

We have recently improved findability, accessibility, and reusability of datasets with COINSTAC with the creation of “vaults”. These are COINSTAC instances set up on cloud or on-premise hardware to facililate federated analysis of sensitive datasets without manual intervention. The data owners can approve only the computations with which they are comfortable or that are appropriate for their data and the variables that they allow to be used in federated analyses (e.g., gender, age, diagnosis). These instances can be found by other users on COINSTAC, added to new consortia, and take part in federated analyses without a need for further approvals and scheduling with the data owners. These vaults will be findable via the Discovery feature and API mentioned above, allowing for improved accessibility. Additionally, with the removal of hurdles to analyze data, we predict that the data will be reused more frequently as well.

### Limitations and Recommendations

We have already highlighted several limitations of the ENIGMA + COINSTAC joint effort in implementing FAIR principles. A critical one for FAIR is the need for persistent, unique identifiers for datasets (or at least their metadata), the pipelines (or again, their structured metadata regarding what was done and how), and the analyses. This is a deficit that we are in the process of remedying, through collaborations with other efforts using identifiers for data that have restricted access, for metadata, and for pipelines, as well as results.

COINSTAC includes instructions for pipeline developers, to aid in integrating their pipeline containers into the COINSTAC platform. Meta-data on what the pipeline does, what parameters are chosen, what software versions were included, and such details, are critical for later re-use and reproducibility, and incorporating structures like the NIDM (Neuroimaging Data Model) (Keator et al., 2013) to describe neuroimaging analyses would be a key step toward improving Reuse. Standards for describing and parameterizing each pipeline will be an active area of development going forward.

Pipelines as containers have numerous advantages, for robustness and reproducibility. In a decentralized environment with privacy concerns, however, privacy protection steps must be included (Mothukuri et al., 2021). The pipelines developed with COINSTAC today have been built to include differential privacy and other approaches to avoid inadvertent data sharing or malicious privacy attacks (Plis et al., 2016; Saha et al., 2020; Sarwate et al., 2014). As the number of pipelines grows, pipeline developers must be aware of the need to ensure that their pipeline isn’t sending data points between sites and to incorporate privacy protection.

COINSTAC shares the limitations of any distributed analysis in assessing data quality. Whether the data were originally high quality but were manipulated incorrectly through the automated analysis pipeline, or whether the data were originally full of errors, methods need to be included to assess the quality (e.g., Glover et al., 2012; Kim et al., 2019)). Pipeline developers can include those options based on best practices for their analyses at the time, and some of the currently available pre-processing pipelines include recommended quality assurance metrics. COINSTAC also supports a computation specifically to help users spot outliers graphically, called dSNE (Saha et al., 2020). dSNE is a powerful method for visualizing large but decentralized datasets and identifying trends, particularly data points which fall outside of the larger groupings.

What COINSTAC does not do, at the moment, is by default check for consistency in *measurement techniques* across sites—if one site measures age in years and another measures it in months, that will not be be useful in a combined analysis without taking that difference into account. If two sites used different software to extract brain measures, that may present a problem for a particular analysis. That is always true in multi-dataset analyses; ENIGMA solved it by allowing each project leader or team to decide what variables they wanted set up in which way for their analysis (e.g. organized in a particular order and named using specific strings in a csv file, or imaging measures which had been processed using only particular software packages and versions), and participating sites who had the needed variables and measures would set them up accordingly for that analysis. COINSTAC currently assumes that the consortium leader will do the same in setting up the analysis pipeline, and that consortium participants whose datasets are being included will have the specified variables and conform to any analysis specifications. Pipeline developers are recommended to include checks for outliers in either the original data or the summary statistics or partial results being shared with the lead site.

A future goal would be to incorporate semantic links, so that, for example, Age in Years as a variable can be distinguished automatically from Age in Months, or that the data provenance specifies the manipulations that have been applied to the imaging or other data follow specifications in the analysis pipeline using standard models (Keator et al., 2013). Through several collaborations, we are taking steps towards solving this problem in COINSTAC by including a detailed description of the subject metadata (e.g., the BIDS participants.json file) in our Discovery feature, which can include links to more detailed ontologies online. As noted above, solving this problem across data, analyses, and results, is clearly a larger and more complex problem that requires collaboration with many other projects.

## Conclusion

This study describes an initial successful multi-center federated meta-analysis automated using COINSTAC. The approach described addresses compromises between access and protection for datasets, allows for simple interoperability without requiring the development of fully semantic annotation, and facilitates data re-use. We believe that federated analysis platforms, such as COINSTAC, will play an increasingly important role in advancing data analyses across federated data sources as they allow for analysis of mixtures of FAIR data along with data that may otherwise not be accessible due to regulatory or other restrictions.

### Information Sharing Statement

COINSTAC is hosted at github (https://github.com/trendscenter/coinstac) with example instructions for its use here (https://github.com/trendscenter/coinstac-instructions). The code for this analysis is at https://github.com/trendscenter/coinstac-enigma-sans, with the Docker image here:

https://hub.docker.com/r/coinstacteam/enigma-sans

ENIGMA analysis protocols for different neuroimaging modalities are available at http://enigma.ini.usc.edu/protocols/.
